# Older Age Is Associated with Fewer Depression and Anxiety Symptoms Following Extreme Weather Adversity

**DOI:** 10.3390/ijerph22101548

**Published:** 2025-10-11

**Authors:** JoNell Strough, Ryan Best, Andrew M. Parker, Esha Azhar, Samer Atshan

**Affiliations:** 1 Department of Psychology, West Virginia University, Morgantown, WV 26506, USA; ryan.best@mail.wvu.edu (R.B.); ea00066@mix.wvu.edu (E.A.); 2 RAND Corporation, Pittsburgh, PA 15213, USA; parker@rand.org; 3 Center for Economic and Social Science Research, University of Southern California, Los Angeles, CA 90089, USA; atshan@usc.edu

**Keywords:** climate change, environmental disaster, resilience, mental health, health, well-being, aging, extreme weather, disaster recovery

## Abstract

Climate change is associated with an increase in the frequency of extreme weather that threatens emotional well-being, with some research pointing to increased vulnerability among older adults. We investigated how age relates to depression and anxiety following adversities due to extreme weather or natural disaster. Socioemotional selectivity theory (SST) posits that older age buffers against emotional distress. The strength and vulnerability integration model (SAVI) posits that this age-related advantage is attenuated during periods of acute stress. Members (*n* = 9761, *M* age = 52.22, *SD* = 16.36 yrs) of a nationally representative, probability-based US internet panel, the Understanding America Study (UAS), reported their experience with extreme weather or natural disaster (e.g., severe storms, tornado, flood), associated adversities (e.g., property loss), and depression and anxiety over the past month. Of the 1075 respondents experiencing extreme weather or natural disaster, 216 reported related adversity. Those experiencing adversity reported more anxiety and depression than those with no events, while extreme weather or disaster alone made no significant difference. Consistent with SST, older age was associated with less depression and anxiety. This age-related benefit was most apparent among those experiencing weather- or disaster-related adversity, even when controlling for socio-demographic correlates. Findings highlight age-related emotional resilience with implications for climate change policy and practice.

## 1. Introduction

Climate change has increased the frequency and severity of extreme weather that threatens public health [1,2,3,4,5]. Extreme weather events such as hurricanes and tornados place adults ages 65 and older at increased risk of mortality due to chronic health conditions and limitations in mobility [6]. Some reviews have argued that disastrous weather also disproportionately disadvantages older adults’ mental health [7,8,9], but others have suggested that older age is protective [10]. A meta-analysis of six studies found that older age increased the risk of post-traumatic stress disorder (PTSD) and adjustment disorder following earthquakes and tsunamis, but was unrelated to depression and anxiety [11]. Other research found that older age was associated with fewer symptoms of depression and PTSD after hurricanes and floods [12,13,14,15].

In the current study, we drew from two prominent theories of aging and emotional well-being, socioemotional selectivity theory (SST) [16], and the strength and vulnerability integration model (SAVI) [17], to investigate potential age differences in the impact of extreme weather or natural disaster and associated adversities on depression and anxiety. The two theories offer contrasting predictions about age differences in emotional resilience. As such, they are well-suited to offer new insights regarding age differences in emotional well-being following climate events. In our investigation, we used data from a national U.S. sample of adults who had or had not experienced a severe storm, tornado, flood, or another extreme weather event or natural disaster in the past month. Much of what is currently known about aging and post-disaster well-being is based on hurricanes [12], floods [14], and earthquakes [18]. By considering a broader array of events, we aimed to expand knowledge to climate events that are increasing with global warming such as storms, droughts, floods, and heatwaves [19].

Aside from inconclusive findings for age differences in post-disaster well-being, cross-sectional and longitudinal research has found that positive emotions increase and negative emotions decrease with age [20,21,22,23,24]. Researchers have labeled this the “well-being paradox” to capture the unexpected nature of age-related improvements occurring alongside decreased physical functioning and other losses [25]. Epidemiological studies found that mood and anxiety disorders were less prevalent in U.S. adults 65 and older compared to middle-aged adults [26,27]. This was also apparent in Western, industrialized countries during the coronavirus disease 2019 (COVID-19) pandemic [28,29,30,31,32,33]. However, not all evidence has been consistent. In the U.S., men aged 70 and older have the highest suicide rate [8,34] and a meta-analysis based on data from several countries indicated that the likelihood of depression increased from age 55 to 89 [35].

Socioemotional selectivity theory (SST) posits that benefits of aging for emotional well-being stem from an increasing awareness that future time left in life is limited which shifts motivation away from the future-oriented goals commonly pursued earlier in life towards pursuing emotional meaning in the “here and now” [16]. Around 60 years of age, perceptions of a limited future overshadowed views of future opportunities [36]. In line with SST, when future time perspective was experimentally limited, individuals prioritized their emotional well-being by, for example, attending more to positive than negative information, choosing to spend time with loved ones, and capitalizing on appealing opportunities [37,38,39].

SST predicts robust benefits of aging for emotional well-being so long as motivation to pursue emotional meaning remains intact [16]. The COVID-19 pandemic was more disruptive to younger adults’ goals than to goals pursued by older adults [40], and this may explain why older adults experienced less psychological distress [31]. Sociocultural events that threatened mortality, including the SARS epidemic and the September 11 attacks, heightened the pursuit of emotional meaning among adults of all ages [41]. Similarly, extreme weather may threaten mortality and bolster older adults’ motivation to seek emotional meaning while disrupting younger adults’ future-oriented goals. Indeed, at least one study found a stronger association between older age and reporting fewer depression symptoms after a hurricane for those who had an associated adversity versus those who had not [15]. Because few studies have considered that exposure to adversity may moderate age differences in post-disaster emotional well-being, the generalizability of this finding is unknown.

The SAVI model argues that age-related emotional benefits stem mainly from avoidance of known stressors [17]. Sustained unavoidable stressors are thought to temporarily diminish age-related advantages for emotional well-being due to physiological vulnerabilities that impair downregulation of negative arousal. Accordingly, during periods of acute stress, between-person age differences in emotional well-being that favor older adults are expected to attenuate or disappear while within-person decreases in emotional well-being are expected to be more pronounced at older ages [42]. In line with SAVI, older age was associated with less negative affect when an interpersonal stressor was avoided, but not when a stressor was encountered [43]. Pandemic stressors increased negative affect for both older and younger adults [44].

Consistent with SAVI, research found worse emotional well-being in older adults after sudden-onset disasters like earthquakes [11], although not all evidence has been consistent [18]. Research inconsistent with SAVI was based mostly on hurricanes in the U.S. Gulf Coast, a region accustomed to dealing with seasonal hurricanes [12,15]. Prior stressful experiences can inoculate against distress when similar challenges re-occur [45]. Older adults on the Gulf Coast may have drawn upon their life experience with hurricanes to cope [46]. It remains unclear if older age is associated with emotional resilience in regions less prone to extreme weather. Climate change may introduce new stressors for such individuals [47].

Psychological distress has been found to spike after a traumatic event and then abate [48]. Most research on age differences has investigated emotional well-being several months, or even years, post-disaster [11]. The heightened vulnerability of older adults to unavoidable stressors predicted by SAVI may be more evident when emotional well-being is assessed sooner after the stressful event [42].

Sociodemographic characteristics related to age, including poorer perceived health [49], increase the risk of emotional distress after a disaster [15,50]. Furthermore, older adults describe chronic health problems as a factor that increases their vulnerability to climate stressors [47]. Taking perceived health into account may thus facilitate a better understanding of the association between extreme weather events and age differences in emotional well-being.

In our study, we used self-reported symptoms of depression and anxiety as indicators of emotional well-being. Experiencing less negative mood is one component of subjective well-being, also referred to as hedonic well-being [51,52]. Our research question asked: After extreme weather or natural disasters, are there age-related differences in depression and anxiety that depend on exposure to related adversity, after accounting for perceived health? Drawing from SST [16], we hypothesized that older age would be associated with fewer depression (Hypothesis 1a) and anxiety (Hypothesis 1b) symptoms. Drawing from SAVI [17], we hypothesized that among those who had experienced weather- or disaster-related adversity, the association between older age and fewer depression (Hypothesis 2a) and anxiety (Hypothesis 2b) symptoms would be weaker. Drawing from research on age differences in post-disaster emotional well-being [15], we considered the alternative hypothesis that among those who had experienced weather- or disaster-related adversity, the association between older age and fewer depression (Hypothesis 3a) and anxiety symptoms (Hypothesis 3b) would be stronger.

## 2. Materials and Methods

### 2.1. Data

Data were from the Understanding America Study (UAS), a nationally representative, probability-based online panel managed by the University of Southern California [53]. The UAS recruits adults aged 18 and above from US households via address-based sampling. To minimize selection bias, individuals who lack connectivity are provided with a 4G Android tablet or an iPhone, and a data plan. Survey weights are available to demographically align the survey sample with the US population. Panel members consent to participate in UAS surveys and for their anonymized data to be used by researchers. All surveys are reviewed and approved by USC’s human subjects committee internal review board before they are distributed. The UAS has been operational since 2014, and had 14,588 active panel members at the time of data collection. UAS surveys cover many topics, including but not limited to finances, consumer behavior, elections, and physical and mental health. Deidentified UAS data is freely available to users who create an account.

The current study used data from UAS Survey 618 which was in the field from 1 May 2024 to 31 May 2024. The survey was sent to all active panelists, 9880 of whom completed it for a completion rate of 67.7%. Per the UAS standard payment of $2 per 3 min of survey time, respondents received $5 for completing the survey. The survey included questions on health, income, and subjective well-being, among others. Survey questions relevant to the current report were those that assessed symptoms of depression and anxiety, perceived health, and panelists’ experience with extreme weather or natural disaster and associated adversities during the past 30 days. Demographic information was collected as part of a separate survey. Of the 9880 panelists that finished the survey, 119 (1.2%) were missing data for at least one of the study variables or covariates. Imputing data for missing values did not influence results (Supplemental Table S1), so the reported results reflect a final sample of 9761 after list-wise deletion. For all reported regression analyses, sampling weights provided by the UAS were incorporated to produce nationally representative estimates.

### 2.2. Measures

#### 2.2.1. Extreme Weather Events or Natural Disasters

Respondents were first asked, “In the past thirty days, did you experience any extreme weather events (for example, extreme heat or cold, severe storm, smoke from wildfire) or natural disaster (for example, hurricane, tornado, tropical storm, wildfire, earthquake, landslide or mudslide, drought, flood, volcanic eruption, tsunami)?” and asked to indicate “yes” or “no.” For those who answered “yes,” a follow-up question asked, “Which of the following extreme weather events or natural disasters did you experience over the last 30 days?” Respondents were asked to indicate all events that applied to them from a list of 15 events (e.g., severe storm, tornado, flood, earthquake), including events other than those listed. A text box was provided for respondents to specify the other event they had experienced, if any. Text box responses (*n* = 73), included 59 extreme weather or natural disaster events that matched one or more of the 15 existing categories and four events (winter storm) that did not match an existing category. Events were reclassified into either an existing category or an “other” category. Irrelevant responses were excluded. Prior to reclassification of responses, E. Azhar and A. Parker independently reviewed responses from a different data set and achieved consensus on parameters for reclassification of textbox responses.

#### 2.2.2. Adverse Consequences

Respondents who indicated that they had experienced extreme weather or natural disaster were asked, “Did you experience any negative consequences of the extreme weather event(s) or natural disaster(s)?” and to indicate, “yes” or “no.” For those answering yes, a follow-up question asked, “Which of the following consequences of the extreme weather event(s) or natural disaster(s), if any, did you experience? Please check all that apply.” Respondents were provided with a list of 16 consequences (e.g., property loss, health problems, unable to go outdoors), including consequences other than those listed. A text box was provided for respondents to specify the other negative consequences they had experienced, if any. Text box responses (*n* = 94) included 47 consequences that matched one or more of the 16 existing categories and 59 consequences that did not match an existing category. Consequences were reclassified into either an existing category or an “other” category. Irrelevant responses were discarded. Prior to reclassification, E. Azhar and A. Parker reviewed 107 responses from different dataset to establish parameters for reclassification of textbox responses. Inter-rater agreement on reclassifications was acceptable. Kappa coefficients ranged from 0.66 to 1.0, with an average value of 0.81.

#### 2.2.3. Anxiety

To assess symptoms of anxiety, we used the two anxiety items from the validated 4-item Patient Health Questionnaire (PHQ-4), a short diagnostic tool which assesses anxiety and depression symptoms in patients [54,55]. Respondents indicated how often over the past 14 days they had been bothered by problems of feeling “nervous, anxious, or on edge” and “not being able to stop or control worrying” on a scale ranging from 1 = Not at all to 4 = Nearly every day. A continuous score was computed by summing the two items; higher scores indicated more anxiety symptoms. The two anxiety items on the PHQ-4 are from the Generalized Anxiety Disorder 2-item (GAD-2) measure which is a short form of the Generalized Anxiety Disorder 7-item (GAD-7) scale developed to assess Generalized Anxiety Disorder in adult primary care patients in clinics [56]. GAD-2 is efficient in assessing anxiety symptoms in primary care patients and shows high sensitivity and specificity [57].

#### 2.2.4. Depression

To assess symptoms of depression, we used the two depression items from the PHQ-4. Respondents indicated how often over the past 14 days they had been bothered by problems of “feeling down, depressed, or hopeless” and “little interest or pleasure in doing things” on a scale ranging from 1 = Not at all to 4 = Nearly every day. A continuous score was computed by summing the two items; higher scores indicated more depression symptoms. The two depression items on the PHQ-4 are from the 2-item Patient Health Questionnaire, which is a short form of the 9-item Patient Health Questionnaire for diagnosing depression in primary care patients [58]. The PHQ-2 was developed as a quick and brief assessment of depression symptoms for patients in a clinical environment [59].

#### 2.2.5. Perceived Health

We used the single item self-assessment of health from the Health and Retirement Study to assess perceived health [60]. Respondents were asked, “Would you say your health is excellent, very good, good, fair, or poor?” and responded on a scale where 1 = excellent, 2 = very good, 3 = good, 4 = fair, and 5 = poor. Scores were reverse coded for the analyses so that higher scores indicated better perceived health.

### 2.3. Analytic Approach

Analyses were conducted using SPSS version 27 and R. Before testing our hypotheses, we examined normality of distributions and computed correlations to identify potential covariates. We computed descriptive statistics to determine the frequency of extreme weather events and natural disasters, and associated adversities, if any, reported by participants. Based on these responses, participants were classified into one of three event groups: (1) a no-event group who did not experience extreme weather or natural disaster, and hence, no related adversity, (2) a weather group who experienced extreme weather or natural disaster, but no related adversity, and (3) an adversity group who experienced at least one adversity as a consequence of extreme weather or natural disaster.

To test our hypotheses regarding age-related reductions in depression (Hypothesis 1a) and anxiety (Hypothesis 1b), and moderation of age differences in depression and anxiety by event group (Hypotheses 2a, 3a and Hypotheses 2b, 3b, respectively), we used the “survey” package in R [61] to estimate two survey-weighted regression equations using Taylor linearization to compute robust standard errors, one for anxiety, and one for depression, with list-wise removal of missing cases. A second set of like models were fit to the data to test the interaction between age, measured as a continuous variable, and event group, measured as a categorical variable with three levels. Event group was dummy coded so that the no-event group was the reference group. Two dummy variables were created to separately compare the weather and adversity groups to the no-event group. Follow-up tests of significant interactions examined the conditional effect of age for each of the three groups.

## 3. Results

### 3.1. Sample Description

Table 1 presents the demographic characteristics of the sample. Participants ranged in age from 18 to 102 years of age (*M* = 52.22, *SD* = 16.36). Sixty percent self-identified as women, 73% as white, and 14.2% as Hispanic or Latino. Detailed information regarding the racial and ethnic composition of the sample, their household income, and education are shown in Table 1.

### 3.2. Preliminary Analyses

The indicators of emotional well-being, symptoms of depression and anxiety, were positively skewed and leptokurtic. Analyses based on a logarithmic transformation of depression and anxiety yielded the same results as non-transformed variables. Reported results are based on non-transformed variables.

Though only 1.2% of data were missing, missingness in study variables was analyzed for patterns. Little’s MCAR test indicated that the missing data was not missing completely at random, χ^2^(173), *p* = 0.02. Follow-up analyses were conducted to investigate possible patterns in missingness. For event group and perceived health, missingness varied significantly by age. Participants with non-missing data for event group reported a mean age of 52.2 years, *SD* = 16.4, compared to 35.9 years, *SD* = 11.6 for those missing this variable, *p* = 0.001. Similarly, participants providing a perceived health rating reported a mean age of 52.2 years, *SD* = 16.4, compared to 34.6 years, *SD* = 12.0 for those missing this variable, *p* = 0.001. To test robustness of the results, missing data was replaced through multiple imputation using the “mice” package in R. The weighted regressions in the supplement were pooled across 20 imputed data sets (Supplemental Table S1), but the pattern of results was not affected. Reported analyses reflect list-wise deletion of missing data.

### 3.3. Correlation Analyses of Study Variables and Demographic Variables

Table 2 shows bivariate correlations between key study variables and demographic variables. Older age and better perceived health were each associated with reporting fewer symptoms of anxiety and depression. Race, income, education, gender, and marital status were correlated with key study variables of anxiety, depression, age, and perceived health. We thus included these variables as covariates in the regression analyses.

### 3.4. Prevalence of Extreme Weather Events or Natural Disaster

Table 3 lists the extreme weather events or natural disasters that respondents reported they had experienced in the last 30 days. Of the 1075 (11%) respondents who reported experiencing an extreme weather event or natural disaster, severe storm was reported by the majority (*n* = 684), followed by extreme heat (*n* = 222), tornado (*n* = 213), earthquake (*n* = 123), flood (*n* = 123), extreme cold (*n* = 117), and other types of events.

### 3.5. Prevalence of Adverse Consequences

Table 4 lists the negative events that respondents reported they had experienced in the past 30 days as a consequence of extreme weather or natural disaster. Of the respondents who reported experiencing extreme weather or natural disaster, 26% (2.8% of the total sample) reported experiencing an adverse consequence of extreme weather or natural disaster. The most commonly reported adverse consequence was loss of property or possessions (*n* = 115).

### 3.6. Regression Analysis of Depression

Table 5 summarizes the regression analysis predicting depression. This analysis tested Hypothesis 1a, that older age would be associated with reporting less depression, Hypothesis 2a, that the association between older age and less depression would be weaker in the group experiencing weather- or disaster-related adversity, and the alternative, Hypothesis 3a, that the association between older age and less depression would be stronger in the adversity group. Perceived health, income, gender, race, education, and marital status were covariates in the analysis.

**Figure 1:**
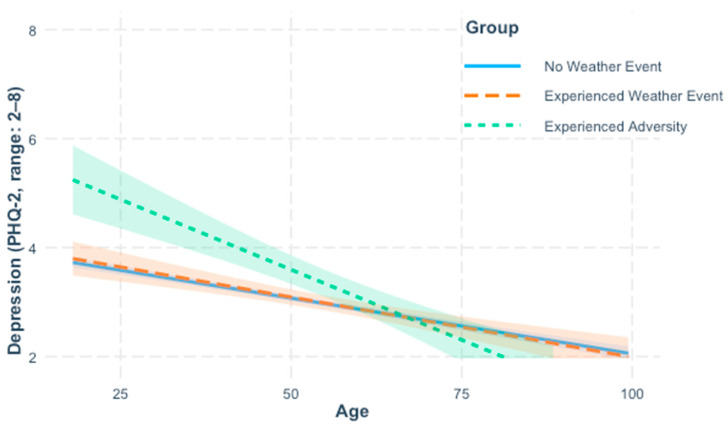
Self-reported depression symptoms by age and event group. Lines reflect effects calculated from regression models controlling for perceived health, income, gender, race, education, and marital status. Shaded areas around lines indicate 95% confidence intervals computed using design-based robust standard errors computed via Taylor linearization using the “survey” package in R (https://www.r-project.org/) to account for survey weights.

In accord with Hypothesis 1a, older age was significantly associated with reporting fewer symptoms of depression, *β* = −0.23, *b* = −0.02, *p* < 0.001. Event group comparisons indicated that the adversity group (M_raw_ = 3.74, SD = 1.92) reported significantly more symptoms of depression than the no-event group (M_raw_ = 2.96, SD = 1.4), *β* = 0.43, *b* = 0.56, *p* < 0.001; the weather group (M_raw_ = 3.07, SD = 1.52) did not differ significantly from the no-event group, *β* = 0.08, *b* = 0.02, *p* = 0.80. Including the interaction term between age and group significantly improved model fit as shown by a survey-weighted likelihood ratio test, *2logLR* = 15.07, *p* = 0.001, indicating that the strength of the association between age and depression differed by group (see Figure 1 and Table 6). Inconsistent with Hypothesis 2a, but in line with alternative Hypothesis 3a, the interaction term between age and the dummy coded weather group variable was non-significant, *β* = −0.02, *b* = −0.002, *p* = 0.67, but the interaction term between age and the dummy coded adversity group variable was significant, *β* = −0.34, *b* = −0.03, *p* < 0.001. Conditional effects showed that the association between older age and less depression was stronger in the adversity group (*b* = −0.05, *p* < 0.001), than the weather group (*b* = −0.02, *p* < 0.001) and the no-event group (*b* = −0.02, *p* < 0.001). That is, older age was associated with reporting fewer depression symptoms across all three groups, but the strength of this association was strongest within the adversity group. Quadratic effects of age and their interactions with event group were tested in additional models, but the results were not significant (Supplemental Table S2).

For the covariates, better perceived health was associated with reporting fewer symptoms of depression, as was higher income, being male, being married, and identifying as non-White. Education was not a significant predictor. Because dichotomization of covariates may obscure important patterns, we re-analyzed the data using the original, non-dichotomized, categorization of covariates. Including non-dichotomized covariates into the models did not change the overall pattern of results (Supplemental Table S3). Likewise, the pattern of results was not affected when running the analyses in unweighted OLS regression models (Supplemental Table S4) or in survey-weighted models estimated with normal standard errors (Supplemental Table S5).

### 3.7. Regression Analysis of Anxiety

Table 5 shows the results of the regression analysis predicting anxiety which tested Hypotheses 1b, 2b, and 3b. Perceived health, income, gender, race, education, and marital status were covariates.

**Figure 2:**
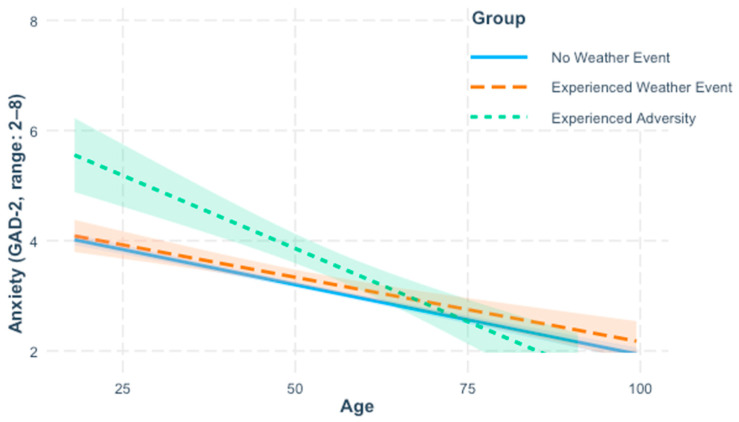
Self-reported anxiety symptoms by age and event group. Lines reflect effects calculated from regression models controlling for perceived health, income, gender, race, education, and marital status. Shaded areas around lines indicate 95% confidence intervals computed using design-based robust standard errors computed via Taylor linearization using the “survey” package in R to account for survey weights.

In line with Hypothesis 1b, older age was significantly associated with reporting fewer symptoms of anxiety, *β* = −0.28, *b* = −0.03, *p* < 0.001. Event group comparisons indicated that the adversity group (*M*_raw_ = 3.92, *SD* = 1.93) reported significantly more symptoms of anxiety than the no-event group (*M*_raw_ = 3.07, *SD* = 1.51), *β* = 0.44, *b* = 0.69, *p* < 0.001; the weather group (*M*_raw_ = 3.25, *SD* = 1.56) did not differ significantly from the no-event group, *β* = 0.08, *b* = 0.13, *p* = 0.07. Including the interaction term between age and group significantly improved model fit as shown by a survey-weighted likelihood ratio test, *2logLR* = 11.84, *p* = 0.002, indicating that the strength of the age–anxiety association varied by group (see Figure 2 and Table 6). Inconsistent with Hypothesis 2b, but in line with alternative Hypothesis 3b, the interaction term between age and the dummy coded weather group variable was non-significant, *β* = 0.02, *b* = 0.002, *p* = 0.61, but the interaction term between age and the dummy coded adversity group variable was significant, *β* = −0.30, *b* = −0.03, *p* = 0.001. Conditional effects showed that the association between older age and less anxiety was strongest in the adversity group (*b* = −0.05, *p* < 0.001), and was significant, though weaker, in the weather group (*b* = −0.02, *p* < 0.001) and the no-event group (*b* = −0.03, *p* < 0.001). Quadratic effects of age and their interactions with event group were tested in additional models, but the results were not significant (Supplemental Table S2).

Similarly to the model predicting depression, better perceived health, higher income, male gender, being married, and non-White racial identity were each associated with reporting fewer symptoms of anxiety. Education was not a significant predictor. Including non-dichotomized covariates into the models did not change the overall pattern of results (Supplemental Table S3). Likewise, the pattern of results was not affected when running the analyses in unweighted OLS regression models (Supplemental Table S4) or in survey-weighted models estimated with normal standard errors (Supplemental Table S5).

## 4. Discussion

We used data from a national U.S. sample of adults who had or had not experienced extreme weather or natural disaster to test theory-driven hypotheses about age differences in emotional resilience in the face of climate change. SST points to age-related motivational shifts to predict robust advantages of aging for emotional well-being [16]. SAVI predicts that sustained unavoidable stressors will attenuate age-related benefits [17]. Consistent with SST, we found that older age was associated with reporting fewer symptoms of depression and anxiety. In contrast to SAVI, these benefits of aging were not weaker among individuals who had experienced adversity due to extreme weather or natural disaster. Instead, age-related reductions in reported symptoms of anxiety and depression were even stronger among individuals who had experienced weather- or disaster-related adversity, confirming predictions based on prior research [15].

The extreme weather events or natural disasters and adversities reported by individuals in our study were diverse, with severe storms being the most common event and property loss the most common adversity. Individuals who experienced adversities as a consequence of extreme weather or natural disaster reported more depression and anxiety symptoms, highlighting the mental health costs of extreme weather, and by extension climate change. Prior research showed similar negative impacts for mental health following hurricane-related adversities [13,15]. The generalizability of these findings to the diverse events reported in our study suggest that the increasing prevalence of extreme weather with climate change [5,19] may further worsen already increasing rates of depression and anxiety in the U.S. [62].

Older age was associated with reporting fewer symptoms of depression and anxiety, echoing findings from a large body of research on aging and emotional well-being [23]. Our findings highlight the robustness of these benefits, with stronger age-related reductions in anxiety and depression in the adversity group compared to the weather and non-event groups. SST attributes well-being benefits of aging to life-span increases in motivation to pursue emotional meaning [16]. Facing adversities due to extreme weather or natural disaster may bolster a focus on emotional meaning, consistent with past findings that mortality-threatening events—such as the SARS epidemic and the September 11 attacks—heightened pursuit of emotionally meaningful goals across adulthood [41]. As during the COVID-19 pandemic [40], facing weather-related adversities may have disrupted younger adults’ pursuit of future-oriented goals, dampening their emotional well-being.

The SAVI model links emotional well-being benefits of aging to stressor avoidance and predicts that unavoidable stressors attenuate age-related advantages for emotional well-being [17], but this was not evident. SAVI acknowledges that older adults’ greater life experience facilitates their emotional well-being, and life experience may have inoculated older adults against weather- or disaster-related adversity [14]. SAVI also emphasizes that age-related vulnerabilities are localized to episodes of acute distress [42]. Our retrospective methods may have contributed to our results since any distress associated with adversity likely faded over time. When reflecting on past events, older adults tend to recall more positive than negative information [63]. They also ruminate less about negative events [64,65] and use positive reappraisal more [66]. Together, these factors may help to explain why older age was associated with reporting less distress following exposure to weather- or disaster-related adversities.

### 4.1. Implications for Policy and Practice

Exposure to adversity related to extreme weather or natural disaster was associated with increased symptoms of depression and anxiety, emphasizing the growing need for mental health services in the face of climate change [3]. Communities can strengthen climate adaptation efforts by integrating psychological services into their plans, for example, by training first responders in psychological first-aid [67]. Expanding access to greenspace may also buffer the mental health impacts of climate change, while simultaneously promoting appreciation for natural environments and motivating climate action [68,69,70]. Embedding mental health care in primary care, telemedicine, and computer-based cognitive behavioral therapy, are ways to expand access to treatment [71,72,73]. Along with expanded access, reducing stigma is key so that those who could benefit from care seek it [74].

Although older age was associated with reporting fewer anxiety and depression symptoms, natural disasters are known to increase older adults’ mortality risk [6]. Many older adults recognize that chronic health problems increase their vulnerability to climate-related stressors [47], but they may underestimate their risk, lack knowledge of how to prepare, and be less likely than younger adults to make emergency plans [75,76]. Emergency preparedness communications for older adults may be more effective when they appeal to emotional meaning [77] and are delivered through preferred formats, such as physical mail rather than email [76].

### 4.2. Limitations and Future Research

Like all studies, our study must be understood in light of its limitations. Individuals who experienced severe adversities following extreme weather or natural disaster may have been unable or unwilling to respond to the survey. Those who did respond reported adversities experienced within the past 30 days and depression and anxiety symptoms within the past 14 days. This means that any symptoms that were present but abated before this 14-day window were not captured. Similarly, if adversities occurred prior to the 30-day window but symptoms persisted, this was not represented in our data. As such, our results may underestimate the association between adversities and emotional well-being. In addition, there was considerable heterogeneity in the severity of adversities reported by individuals. Further research is necessary to understand the extent to which event severity moderates our findings. In such investigations, it will be important to account for individual differences in appraisals of adversities which have been shown to vary by age [78]. Furthermore, our findings based on a U.S. national sample may not generalize to populations outside of the U.S. due to regional differences in vulnerability to climate events, access to healthcare, and cultural differences in strategies for coping with adversity [79,80].

One challenge to understanding the consequences of extreme weather or natural disaster is identifying and contacting individuals who have experienced an event. Much research has targeted survivors after a hurricane, flood, or earthquake and measured their well-being several months or even years afterwards [11]. Our study used a different approach by surveying members of an existing national panel and asking them to report events they had experienced in the last 30 days. Our method lacks efficiency since the vast majority of individuals were not exposed to any extreme weather or natural disaster. However, it facilitates collection of data soon after an event and allows diverse events to be investigated. It also captures individuals’ definitions of extreme weather or natural disaster, which may not necessarily match governmental declarations of emergency or disaster. Understanding how individuals define extreme weather and natural disaster may yield new insights about the impact of climate change on emotional resilience.

The correlational, cross-sectional, retrospective design we used precludes causal conclusions. We cannot rule out the possibility that individuals who were less depressed or anxious prior to experiencing extreme weather or natural disaster were less likely to perceive associated consequences as adversities. In addition, the age differences we found may reflect distinct experiences of different birth cohorts rather than maturational change. To disentangle birth cohort and maturation, future research could employ a cohort-sequential design to follow multiple birth cohorts across time [81].

Our data were collected in May. There is substantial seasonable variation in extreme weather and heatwaves were not well-represented in our data. Heatwaves may harm mental health even in the absence of associated adversities. Monthly temperatures above 30 °C have been linked to increased reports of mental health problems [82]. Extreme heat and cold pose significant risks to older adults’ physical health due to deficits in thermoregulation [83]; further research is necessary to understand risks to mental health. Identifying pathways that link extreme heat to mental health problems and how these pathways vary by age is essential for designing interventions to support climate adaptation.

Our results are based on events occurring within a 30-day interval. Cumulative exposure to weather-related adversities over longer intervals may reveal age-related vulnerabilities posited by SAVI when using longitudinal methods to investigate within-person change instead of the between-person differences we investigated [42]. More broadly, studying the consequences of multiple events over time could highlight the link between extreme weather and climate change and shift planning away from reactive emergency response toward climate change adaptation [67].

Although the SAVI model predicts that unavoidable stressors temporarily diminish the emotional well-being advantages of older adults due to age-related physiological vulnerabilities, this process applies specifically to within-person changes during periods of acute stress. Our study, in contrast, examined between-person differences in depression and anxiety symptoms following adversity due to extreme weather or natural disaster. From this perspective, our finding that age-related emotional advantages were strongest among those who experienced adversity appears inconsistent with SAVI’s general implication that older adults are more emotionally vulnerable under stress. It does not, however, directly test SAVI’s within-person prediction that emotional well-being is compromised in the moment of high arousal. Further work using longitudinal or experience sampling methods would be needed to assess this key prediction.

## 5. Conclusions

Climate change and associated increases in the frequency of extreme weather have been shown to heighten older adults’ risk of mortality due to physical limitations and chronic health conditions [6]. Our study investigated whether adversities associated with extreme weather or natural disaster were disproportionately disadvantageous to mental health depending on age. We found that older age was associated with reporting fewer symptoms of anxiety and depression even when facing adversities related to extreme weather or natural disaster, suggesting increased emotional resilience with age. Further research is necessary to determine the generalizability of our results to other extreme weather events that are projected to increase with climate change such as heatwaves [5]. Further research is also necessary to identify mechanisms underlying older adults’ emotional resilience so that interventions can be designed to promote climate adaptation in people of all ages.

## Figures and Tables

**Table 1:** Sample Demographics.

Variable	N	%	M (SD)
Age	9761	--	52.22 (16.36)
Gender			
Female	5848	59.9	--
Male	3913	40.1	--
Marital Status			
Married	5191	53.2	--
Separated	171	1.8	--
Divorced	1436	14.7	--
Widowed	503	5.2	--
Never married	2460	25.2	--
Race			
White	7123	73.0	--
Black	1099	11.3	--
American Indian/Alaskan Native	197	2.0	--
Asian	761	7.8	--
Hawaiian/Pacific Islander	58	0.6	--
Mixed	523	5.4	--
Ethnicity			
Spanish/Hispanic/Latino	1383	14.2	--
Education			
Less than High School graduate	439	4.5	--
High school graduate/GED	1528	15.7	--
Some college/Associate degree	3265	33.4	--
Bachelor’s degree	2556	26.2	--
Master’s degree	1467	15.0	--
Professional school degree	250	2.6	--
Doctorate degree	256	2.6	--
Annual Household Income			
Less than $5000–$12,499	934	9.6	--
$12,500–$29,999	1218	12.4	--
$30,000–$59,999	2170	22.3	--
$60,000–$74,999	919	9.4	--
$75,000–$150,000+	4520	46.3	--

**Table 2:** Correlations Among Study Variables.

Variable	1	2	3	4	5	6	7	8	9	10	11
1. Depression	—	0.75 **	−0.20 **	0.01	0.07 **	−0.38 **	−0.03 **	−0.18 **	−0.12 **	−0.07 **	−0.18 **
2. Anxiety		—	−0.25 **	0.03 **	0.08 **	−0.32 **	−0.03 **	−0.14 **	−0.08 **	−0.13 **	−0.15 **
3. Age			—	0.01	−0.01	−0.05 **	0.16 **	−0.03 **	−0.01	0.12 **	0.14 **
4. Extreme Weather/Disaster				—	—	−0.05 **	0.00	−0.06 **	−0.06 **	−0.01	−0.01
5. Adverse Consequence					—	−0.03 **	−0.03 **	−0.05 *	−0.04 **	−0.01	−0.03 **
6. Perceived Health						—	0.06 **	0.25 **	0.23 **	0.03 **	0.12 **
7. Race							—	0.10 **	0.01	0.05 **	0.16 **
8. Income								—	0.41 **	0.13 **	0.33 **
9. Education									—	0.10 **	0.13 **
10. Gender										—	0.14 **
11. Marital Status											

*N* = 9761, * *p* < 0.05, ** *p* < 0.01; Extreme Weather or Natural Disaster = 1, No Event = 0; Adverse Consequence = 1, No Consequence = 0; Race: White/Caucasian = 1, Not white/Caucasian = 0; Income: Annual household income ≥ $75,000 = 1, annual household income < $75,000 = 0; Education: Bachelor’s degree or greater = 1, less than a Bachelor’s degree = 0; Gender (measured in the UAS as a binary variable): 1 = male, 0 = female; Marital Status: Married = 1, Not Married = 0.

**Table 3:** Extreme Weather Events or Natural Disasters in the Past 30 Days Reported by Survey Respondents.

Event	Count	%
Severe storm	684	63.6%
Extreme heat	222	20.7%
Tornado	213	19.8%
Earthquake	123	11.4%
Flood	123	11.4%
Extreme cold	117	10.9%
Smoke from wildfire	44	4.1%
Tropical storm	26	2.4%
Drought	17	1.6%
Hurricane	14	1.3%
Wildfire	9	0.8%
Other *	4	0.4%
Landslide or mudslide	4	0.4%
Tsunami	2	0.2%
Volcanic eruption	1	0.1%
Any Event	1075	

*N* = 1075. Reports of extreme weather or natural disasters were obtained between 1 May 2024 and 31 May 2024. Percentages were calculated by dividing the number who experienced a given event by the total number experiencing extreme weather or natural disaster. * The “other” events were winter storms.

**Table 4:** Adverse Consequences Reported by Respondents Who Experienced Extreme Weather or Natural Disaster.

Consequence	Count	%
Loss or destruction of property, possessions	115	10.7%
Other *	59	5.5%
Unable to spend time outdoors	55	5.1%
Health problem(s)	41	3.8%
Loss of income due to inability to work	24	2.2%
Temporary evacuation	19	1.8%
Took on additional debt	17	1.6%
Did not have adequate food for your family	16	1.5%
Person you know was injured or killed	12	1.1%
Unable to meet essential expenses	11	1.0%
Unable to get to work (no transportation)	10	0.9%
Pets were injured, killed, or lost	7	0.7%
Unable to obtain medications when needed	5	0.5%
Unable to obtain needed medical care	5	0.5%
Could not find adequate childcare	5	0.5%
At least one consequence	216	

*N* = 216. Some respondents experienced more than one adverse consequence. Percentages were calculated by dividing the number who experienced a given adverse consequence by the number experiencing extreme weather or natural disaster (*n* = 1075). Reports of adverse consequences due to extreme weather or natural disaster were obtained between 1 May 2024 and 31 May 2024. * Of the “other” adverse consequences, the majority were loss of power or other utilities (*n* = 37), followed by damage to infrastructure such as bridges (*n* = 7), flooding of home or roads (*n* = 5), increased utility costs (*n* = 3), general discomfort (*n* = 3), extra expenses (*n* = 2), extra work (*n* = 1), and travel disruption (*n* = 1).

**Table 5:** Weighted Regression Coefficients Predicting Depression and Anxiety from Age, Event Group, and Covariates with Robust Standard Errors.

Predictor	Depression				Anxiety			
	*B*	*SE*	*t*	*p*	*B*	*SE*	*t*	*p*
Constant	6.16	0.12	52.95	<0.001	6.16	0.12	53.21	<0.001
Age	−0.02	<0.01	−16.50	<0.001	−0.03	<0.01	−20.45	<0.001
Adversity vs. No Event	2.08	0.46	4.50	<0.001	2.03	0.49	4.14	<0.001
Weather vs. No Event	0.01	0.23	0.43	0.67	0.03	0.22	0.15	0.88
Age × Adversity	−0.03	0.01	−3.87	<0.001	−0.03	0.01	−3.24	0.001
Age × Weather	<−0.01	<0.01	−0.40	0.69	<0.01	<0.01	0.52	0.61
Health	−0.61	0.02	−24.78	<0.001	−0.52	0.02	−21.46	<0.001
Race	0.16	0.05	3.24	<0.001	0.20	0.05	4.30	<0.001
Income	−0.28	0.04	−6.39	<0.001	−0.20	0.04	−4.65	<0.001
Education	0.03	0.04	0.81	0.42	0.04	0.04	1.07	0.29
Gender	−0.11	0.04	−2.64	0.01	−0.24	0.04	−5.89	<0.001
Marital Status	−0.22	0.04	−5.07	<0.001	−0.16	0.04	−3.74	<0.001

*N* = 9761. Race: white/Caucasian = 1, not white/Caucasian = 0; Income: Annual household income > $75,000 = 1, annual household income <$75,000 = 0; Education: Bachelor’s degree or greater = 1, less than a Bachelor’s degree = 0; Gender (measured in the UAS as a binary variable): male = 1, female = 0; Marital Status: Married = 1, Not married = 0. Design-based robust standard errors computed via Taylor linearization using the survey package in R to account for survey weights.

**Table 6:** Predicted Marginal Means by Age and Event Group from Survey-Weighted Models.

Age	Event Group	Depression		Anxiety	
		EM Mean	95% CI	EM Mean	95% CI
30	No Event	3.35	[3.28, 3.42]	3.51	[3.44, 3.58]
	Weather	3.40	[3.16, 3.63]	3.60	[3.39, 3.82]
	Adversity	4.49	[4.01, 4.96]	4.72	[4.22, 5.21]
50	No Event	2.94	[2.89, 2.98]	3.00	[2.69, 3.05]
	Weather	2.96	[2.82, 3.10]	3.13	[3.00, 3.27]
	Adversity	3.46	[3.20, 3.72]	3.66	[3.39, 3.92]
70	No Event	2.53	[2.47, 2.59]	2.49	[2.43, 2.55]
	Weather	2.51	[2.34, 2.69]	2.67	[2.48, 2.85]
	Adversity	2.43	[2.09, 2.77]	2.60	[2.25, 2.94]

## Data Availability

This research relies on data from surveys administered by the Understanding America Study (UAS), which is maintained by the Center for Economic and Social Research (CESR) at the University of Southern California. De-identified data and codebooks for all surveys are publicly and freely available (https://uasdata.usc.edu URL accessed on 18 August 2025).

## Supplementary Materials

The following supporting information can be downloaded at: https://www.mdpi.com/article/10.3390/ijerph22101548/s1, Table S1: Weighted Regression Coefficients Predicting Depression and Anxiety from Age, Event Group, and Covariates with Robust Standard Errors on 20 Pooled Datasets with Missingness Replaced with Multiple Imputation; Table S2: Weighted Regression Coefficients Predicting Depression and Anxiety from Age, Event Group, and Covariates with Robust Standard Errors including Quadratic Age Terms; Table S3: Weighted Regression Coefficients Predicting Depression and Anxiety from Age, Event Group, and Covariates with Robust Standard Errors included Original Coding of Covariates; Table S4: Regression Coefficients Predicting Depression and Anxiety from Age, Event Group, and Covariates without Survey Weights; Table S5: Regression Coefficients Predicting Depression and Anxiety from Age, Event Group, and Covariates while including UAS Population Weights.

## Abbreviations

The following abbreviations are used in this manuscript:

SAVI	Strength and Vulnerability Integration
SST	Socioemotional Selectivity Theory
