# Supplementary material for: Older Age Is Associated with Fewer Depression and Anxiety Symptoms Following Extreme Weather Adversity
Source: Int J Environ Res Public Health. 2025 Oct 11;22(10):1548. doi: 10.3390/ijerph22101548 (PMC12564414; doi:10.3390/ijerph22101548)
Supplement: Supplementary file 1 [file ijerph-22-01548-s001.zip › ijerph-3855973-supplementary.pdf]

Supplemental Table S1. Weighted Regression Coefficients Predicting Depression and Anxiety from Age, Event Group, and Covariates with Robust Standard Errors on 20 Pooled Datasets with Missingness Replaced with Multiple Imputation

**Predictor**	**Depression**				**Anxiety**			
	B	***SE***	***t***	***p***	***B***	***SE***	***t***	***p***
Constant	6.16	0.07	83.18	< .001	6.17	0.07	82.65	< .001
Age	-0.02	<0.01	-21.86	< .001	-0.03	<0.01	-26.83	< .001
Adversity vs. No Event	2.04	0.30	6.82	< .001	2.01	0.30	6.65	< .001
Weather vs. No Event	0.1	0.15	0.67	0.50	0.11	0.15	0.69	0.49
Age × Adversity	-0.03	0.01	-5.11	< .001	-0.03	0.01	-4.51	< .001
Age × Weather	<–0.01	<0.01	-0.57	0.57	<0.01	<0.01	0.29	0.77
Health	-0.61	0.02	-37.18	< .001	-0.53	0.02	-31.83	< .001
Race	0.16	0.03	4.68	< .001	0.21	0.03	6.21	< .001
Income	-0.27	0.03	-7.79	< .001	-0.19	0.04	-5.5	< .001
Education	0.03	0.03	0.94	0.35	0.04	0.03	1.2	0.23
Gender	-0.11	0.03	-3.63	< .001	-0.24	0.03	-7.99	< .001
Marital Status	-0.21	0.03	-6.76	< .001	-0.16	0.03	-5.18	< .001

N=9,761. Race: white/Caucasian=1, not white/Caucasian=0; Income: Annual household income ≥ \$75,000=1, annual household income <\$75,000=0; Education: Bachelor's degree or greater=1, less than a Bachelor's degree=0; Gender (measured in the UAS as a binary variable): male=1, female=0; Marital Status: Married=1, Not married=0. Missing data was replaced with multiple imputation.

Supplemental Table S2. Weighted Regression Coefficients Predicting Depression and Anxiety from Age, Event Group, and Covariates with Robust Standard Errors including Quadratic Age Terms

**Predictor**	**Depression**				**Anxiety**			
	B	***SE***	***t***	***p***	***B***	***SE***	***t***	***p***
Constant	5.99	0.21	28.42	< .001	6.27	0.21	29.24	< .001
Age	-0.01	0.01	-1.76	0.08	-0.03	0.01	-3.91	< .001
Age^2	<–0.01	<0.01	-1.01	0.31	<0.01	<0.01	0.64	0.52
Adversity vs. No Event	1.07	1.29	0.83	0.41	1.39	1.46	0.95	0.34
Weather vs. No Event	-0.08	0.57	-0.13	0.89	-0.32	0.55	-0.58	0.56
Age × Adversity	0.01	0.05	0.28	0.78	<0.01	0.06	0.02	0.98
Age^2 × Adversity	<–0.01	<0.01	-1.01	0.31	<–0.01	<0.01	-0.58	0.56
Age × Weather	0.01	0.02	0.28	0.78	0.02	0.02	0.81	0.42
Age^2 × Weather	<–0.01	<0.01	-0.39	0.7	<–0.01	<0.01	-0.77	0.44
Health	-0.61	0.02	-24.61	< .001	-0.52	0.02	-21.42	< .001
Race	0.16	0.05	3.31	< .001	0.20	0.05	4.29	< .001
Income	-0.28	0.04	-6.48	< .001	-0.20	0.04	-4.59	< .001
Education	0.03	0.04	0.83	0.41	0.04	0.04	1.05	0.29
Gender	-0.11	0.04	-2.61	0.01	-0.24	0.04	-5.90	< .001
Marital Status	-0.22	0.04	-5.17	< .001	-0.16	0.04	-3.64	< .001

N=9,761. Race: white/Caucasian=1, not white/Caucasian=0; Income: Annual household income ≥ $75,000=1, annual household income <$75,000=0; Education: Bachelor’s degree or greater=1, less than a Bachelor’s degree=0; Gender (measured in the UAS as a binary variable): male=1, female=0; Marital Status: Married=1, Not married=0.

Supplemental Table S3. Weighted Regression Coefficients Predicting Depression and Anxiety from Age, Event Group, and Covariates with Robust Standard Errors included Original Coding of Covariates

**Predictor**	**Depression**				**Anxiety**			
	B	*SE*	*t*	*p*	*B*	*SE*	*t*	*p*
Constant	5.79	0.18	31.74	< .001	5.62	0.2	27.87	< .001
Age	-0.02	<0.01	-15.54	< .001	-0.02	<0.01	-19.57	< .001
Adversity vs. No Event	2.01	0.46	4.35	< .001	2.00	0.48	4.15	< .001
Weather vs. No Event	0.07	0.23	0.32	.75	0.05	0.22	0.22	0.83
Age × Adversity	-0.03	0.01	-3.77	< .001	-0.03	0.01	-3.27	< .001
Age × Weather	<–0.01	<0.01	-0.34	.73	<0.01	<0.01	0.43	0.67
Health	–0.60	0.02	-24.20	< .001	-0.52	0.02	-21.18	< .001
Race								
Black	-0.32	0.07	-4.84	< .001	-0.36	0.06	-5.49	< .001
American Indian or Alaska Native only	-0.09	0.19	-0.5	0.62	-0.23	0.16	-1.45	0.15
Asian only	-0.04	0.08	-0.48	0.63	-0.05	0.08	-0.60	0.55
Hawaiian/Pacific Islander only	-0.20	0.25	-0.78	0.44	-0.54	0.19	-2.86	< .001
Mixed	-0.03	0.09	-0.33	0.74	-0.09	0.09	-0.99	0.32
Income								
$5,000-$7,499	-0.18	0.22	-0.82	0.41	-0.19	0.22	-0.88	0.38
$7,500-$9,999	0.1	0.24	0.42	0.67	0.04	0.24	0.19	0.85
$10,000-$12,499	0.07	0.18	0.38	0.71	0.08	0.18	0.47	0.64
$12,500-$14,999	-0.04	0.22	-0.19	0.85	0.09	0.20	0.45	0.65
$15,000-$19,999	-0.19	0.16	-1.16	0.25	-0.21	0.15	-1.34	0.18
$20,000-$24,999	-0.24	0.16	-1.47	0.14	-0.20	0.15	-1.28	0.2
$25,000-$29,999	-0.33	0.16	-2.12	0.03	-0.28	0.15	-1.9	0.06
$30,000-$34,999	-0.21	0.15	-1.4	0.16	-0.10	0.15	-0.63	0.53
$35,000-$39,999	-0.28	0.15	-1.89	0.06	-0.18	0.15	-1.24	0.21
$40,000-$49,999	-0.38	0.13	-2.85	< .001	-0.31	0.14	-2.27	0.02

Predictor	Depression				Anxiety			
	B	*SE*	*t*	*p*	*B*	*SE*	*t*	*p*
$50,000-$59,999	-0.44	0.13	-3.30	< .001	-0.34	0.13	-2.52	0.01
$60,000-$74,999	-0.35	0.13	-2.65	0.01	-0.31	0.13	-2.35	0.02
$75,000-$99,999	-0.55	0.12	-4.51	< .001	-0.44	0.12	-3.66	< .001
$100,000-$149,999	-0.52	0.12	-4.31	< .001	-0.43	0.12	-3.51	< .001
$150,000 or more	-0.62	0.12	-5.01	< .001	-0.46	0.12	-3.71	< .001
Education								
Up to 4th grade	0.59	0.50	1.17	0.24	0.85	0.45	1.91	0.06
5th or 6th grade	-0.40	0.23	-1.75	0.08	-0.17	0.25	-0.70	0.48
7th or 8th grade	0.96	0.40	2.40	0.02	0.77	0.33	2.36	0.02
9th grade	0.43	0.20	2.11	0.03	0.76	0.24	3.20	< .001
10th grade	0.88	0.21	4.15	< .001	0.98	0.23	4.19	< .001
11th grade	0.69	0.22	3.13	< .001	0.99	0.24	4.17	< .001
12th grade	0.70	0.20	3.58	< .001	0.89	0.22	4.12	< .001
High school graduate or GED	0.70	0.10	6.91	< .001	0.88	0.14	6.42	< .001
Some college/no degree	0.69	0.10	6.74	< .001	0.95	0.14	6.96	< .001
Assoc. college degree-occ/voc prog.	0.59	0.11	5.18	< .001	0.83	0.15	5.65	< .001
Assoc. college degree-academic prog.	0.67	0.12	5.64	< .001	1.02	0.15	6.68	< .001
Bachelor's degree	0.76	0.10	7.59	< .001	0.93	0.13	6.91	< .001
Master's degree	0.70	0.10	7.05	< .001	0.96	0.14	7.08	< .001
Professional school degree	0.75	0.13	5.84	< .001	1.15	0.18	6.58	< .001
Doctorate degree	0.74	0.13	5.57	< .001	1.07	0.17	6.41	< .001
Gender	-0.11	0.04	-2.70	.01	-0.24	0.04	-6.02	< .001

Predictor	Depression				Anxiety			
	B	***SE***	***t***	***p***	***B***	***SE***	***t***	***p***
Marital Status	-0.18	0.04	-4.16	< .001	-0.14	0.04	-3.14	< .001

N=9,761. Reference categories are Race: white/Caucasian; Income: Annual household income <$5,000; Education: less than 1st grade; Gender: female; Marital Status: Not married. Design-based robust standard errors computed via Taylor linearization using the *survey* package in R to account for survey weights.

Supplemental Table S4. Regression Coefficients Predicting Depression and Anxiety from Age, Event Group, and Covariates without Survey Weights

**Predictor**	**Depression**				**Anxiety**			
	B	***SE***	***t***	***p***	***B***	***SE***	***t***	***p***
Constant	6.06	0.07	84.68	< .001	6.16	0.07	84.05	< .001
Age	–0.02	<0.01	–20.86	< .001	–0.02	<0.01	–26.19	< .001
Adversity vs. No Event	1.88	0.31	6.04	< .001	1.76	0.32	5.53	< .001
Weather vs. No Event	0.01	0.16	0.09	.93	0.12	0.17	0.73	.47
Age × Adversity	–0.03	0.01	–4.44	< .001	–0.02	0.01	–3.59	< .001
Age × Weather	<–0.01	<0.01	–0.09	.93	<–0.01	<0.01	–0.21	.83
Health	–0.60	0.02	–38.22	< .001	–0.53	0.02	–32.97	< .001
Race	0.15	0.03	4.68	< .001	0.18	0.03	5.72	< .001
Income	–0.21	0.03	–6.68	< .001	–0.14	0.03	–4.31	< .001
Education	0.01	0.03	0.39	.70	0.07	0.03	2.22	.03
Gender	–0.06	0.03	–2.02	.04	–0.23	0.03	–8.11	< .001
Marital Status	–0.26	0.03	–8.88	< .001	–0.17	0.03	–5.64	< .001

N=9,761. Race: white/Caucasian=1, not white/Caucasian=0; Income: Annual household income ≥ $75,000=1, annual household income <$75,000=0; Education: Bachelor's degree or greater=1, less than a Bachelor's degree=0; Gender (measured in the UAS as a binary variable): male=1, female=0; Marital Status: Married=1, Not married=0.

Supplemental Table S5. Regression Coefficients Predicting Depression and Anxiety from Age, Event Group, and Covariates while including UAS Population Weights

**Predictor**	**Depression**				**Anxiety**			
	B	***SE***	***t***	***p***	***B***	***SE***	***t***	***p***
Constant	6.16	0.07	83.59	< .001	6.17	0.07	83.14	< .001
Age	–0.02	<0.01	–21.82	< .001	–0.03	<0.01	–26.98	< .001
Adversity vs. No Event	2.08	0.30	6.97	< .001	2.03	0.30	6.78	< .001
Weather vs. No Event	0.01	0.15	0.66	.51	0.03	0.15	0.22	.83
Age × Adversity	–0.03	0.01	–5.40	< .001	–0.03	0.01	–4.76	< .001
Age × Weather	<–0.01	<0.01	–0.56	.58	<0.01	<0.01	0.69	.49
Health	–0.61	0.02	–37.13	< .001	–0.52	0.02	–31.73	< .001
Race	0.16	0.03	4.63	< .001	0.20	0.03	6.03	< .001
Income	–0.28	0.03	–7.83	< .001	–0.20	0.03	–5.67	< .001
Education	0.03	0.03	0.95	.34	0.04	0.03	1.24	.22
Gender	–0.11	0.03	–3.64	<.001	–0.24	0.03	–8.05	< .001
Marital Status	–0.22	0.03	–6.88	< .001	–0.16	0.03	–5.11	< .001

N=9,761. Race: white/Caucasian=1, not white/Caucasian=0; Income: Annual household income ≥ $75,000=1, annual household income <$75,000=0; Education: Bachelor’s degree or greater=1, less than a Bachelor’s degree=0; Gender: male=1, female=0; Marital Status: Married=1, Not married=0.
